# Moderate- to high intensity aerobic and resistance exercise reduces peripheral blood regulatory cell populations in older adults with rheumatoid arthritis

**DOI:** 10.1186/s12979-020-00184-y

**Published:** 2020-05-16

**Authors:** Sofia E. M. Andersson, Elvira Lange, Daniel Kucharski, Sara Svedlund, Karin Önnheim, Maria Bergquist, Elisabet Josefsson, Janet M. Lord, Inga-Lill Mårtensson, Kaisa Mannerkorpi, Inger Gjertsson

**Affiliations:** 1 Department of Rheumatology and Inflammation research, Institute of Medicine, The Sahlgrenska Academy, University of Gothenburg, Box 480, 405 30 Göteborg, Sweden; 2 University of Gothenburg Centre for Person-Centred Care, Gothenburg, Sweden; 3 Department of Health and Rehabilitation, Institute of Neuroscience and Physiology, The Sahlgrenska Academy, University of Gothenburg, Gothenburg, Sweden; 4 Department of Molecular and Clinical Medicine, Institute of Medicine, The Sahlgrenska Academy, University of Gothenburg, Gothenburg, Sweden; 5 Department of Medical Sciences, Clinical Physiology, Uppsala University, Uppsala, Sweden; 6 MRC-ARUK Centre for Musculoskeletal Ageing Research, Institute of Inflammation and Ageing, University of Birmingham, Birmingham, UK

**Keywords:** Rheumatoid arthritis, Aging, Exercise, Treg cells, T cells, Breg cells, IL-10

## Abstract

**Objective:**

Exercise can improve immune health and is beneficial for physical function in patients with rheumatoid arthritis (RA), but the immunological mechanisms are largely unknown. We evaluated the effect of moderate- to high intensity exercise with person-centred guidance on cells of the immune system, with focus on regulatory cell populations, in older adults with RA.

**Methods:**

Older adults (≥65 years) with RA were randomized to either 20-weeks of moderate – to high intensity aerobic and resistance exercise (*n* = 24) or to an active control group performing home-based exercise of light intensity (*n* = 25). Aerobic capacity, muscle strength, DAS28 and CRP were evaluated. Blood samples were collected at baseline and after 20 weeks. The frequency of immune cells defined as adaptive regulatory populations, CD4 + Foxp3 + CD25 + CD127- T regulatory cells (Tregs) and CD19 + CD24hiCD38hi B regulatory cells (Bregs) as well as HLA-DR−/lowCD33 + CD11b + myeloid derived suppressor cells (MDSCs), were assessed using flow cytometry.

**Results:**

After 20 weeks of moderate- to high intensity exercise, aerobic capacity and muscle strength were significantly improved but there were no significant changes in Disease Activity Score 28 (DAS28) or CRP. The frequency of Tregs and Bregs decreased significantly in the intervention group, but not in the active control group. The exercise intervention had no effect on MDSCs. The reduction in regulatory T cells in the intervention group was most pronounced in the female patients.

**Conclusion:**

Moderate- to high intensity exercise in older adults with RA led to a decreased proportion of Tregs and Bregs, but that was not associated with increased disease activity or increased inflammation.

**Trial registration:**

Improved Ability to Cope With Everyday Life Through a Person-centered Training Program in Elderly Patients With Rheumatoid Arthritis - PEP-walk Study, NCT02397798. Registered at ClinicalTrials.gov March 19, 2015.

## Background

Rheumatoid arthritis (RA) is an autoimmune joint disease driven by complex immune dysregulation and which may have a profound impact on healthy aging. Having RA accelerates aging [1] and age-related changes in the immune system are believed to increase the risk for autoimmune disease, infections and cancer development [2]. Animal studies suggest that regulatory T cells (Tregs) are less efficient in dampening autoimmune pro-inflammatory responses with age but are on the other hand more prone to cause erroneous protection of cancer cells [3, 4] and allow reactivation of chronic viral infections [5]. With age, Treg frequency is increased in blood and tissues, however it is less clear whether the Tregs function is altered [3, 6]. Patients with RA seemingly have a reduced frequency of Tregs (Foxp3 + CD25+) in peripheral blood compared to healthy individuals, and a higher frequency in synovial fluid compared to peripheral blood [7]. Regulatory CD24hiCD38hi B cells (Bregs) are reduced in patients with active RA [8] and both frequency of Bregs as well as their IL10 production decline with age [9]. Myeloid-derived suppressor cells (MDSCs) are a heterogeneous population of myeloid derived cells at an early stage of development that exhibits immunosuppressive properties and they increase in peripheral blood with age [10] and during chronic inflammatory conditions, such as autoimmune diseases and cancer [11].

Exercise has a revitalizing effect on the aging immune system and promotes healthy immune function, for example it improves the response to vaccines [12], without aggravating autoimmune responses [13]. It promotes acute release of anti-inflammatory myokines from skeletal muscle and causes reduction of visceral fat releasing pro-inflammatory adipokines [13, 14] thereby it reduces the chronic low-grade inflammation known as inflamm-aging [12]. We have shown that older adults (≥65 years) with RA improve significantly with respect to aerobic capacity and muscle strength, fatigue and patient’s global impression of change (PGIC) [15, 16] after 20 weeks of moderate- to high intensity aerobic and resistance exercise with person-centred guidance [15].

However, it is not currently known if moderate- to high intensity exercise in older adults with RA will have an effect on immune regulation. This study aimed to evaluate the effects of a 20 weeks moderate- to high intensity, aerobic and resistance exercise intervention with person-centred guidance on regulatory immune cell populations in patients with RA ≥ 65 years of age. We hypothesized that moderate- to high intensity exercise would influence regulatory immune cell subsets in peripheral blood of older adults with RA.

## Methods

### Participants

This randomized controlled study contains data from the Gothenburg-cohort comprising 49 patients recruited at Sahlgrenska University Hospital, Gothenburg. The Gothenburg-cohort is a subset of a total of 74 patients included in the Pep-walk study [15]. The inclusion criteria were patients diagnosed with RA according to the ACR 1987/EULAR 2010 criteria, age ≥ 65 years, disease duration ≥2 years and DAS28 score < 5.1. The exclusion criteria were other significant comorbidity such as unstable coronary heart disease or dysregulated cardiac arrhythmia that precludes moderate to intense exercise, joint surgery within 6 months prior to inclusion, already on-going exercise on moderate to high level ≥ 2 times week, other factors that would influence the participation such as residence far from site or insufficient communicative abilities. All patients were recruited in 2015 and the samples were collected during 2015 and 2016. Out of the 49 patients recruited at Sahlgrenska University Hospital, Gothenburg, 48 patients completed the study [15]. Patient characteristics are shown in Table 1. Clinical data for all patients (*n* = 74) together is published by Lange et al. [15].

**Table 1:** Baseline demographics and clinical characteristics of participants

	Control (***n*** = 25)	Exercise (***n*** = 24)	***p***-value
Female, n (%)	21 (84%)	19 (79%)	0.73
Mean age (years) ± SD	70 ± 2.4	69 ± 2.7	0.17
Disease duration, median ± IQR	20 (11–24)	13 (9.3–19)	0.06
HAQ-DI, median ± IQR	0.50 (0.25–0.94)	0.19 (0.0–0.84)	0.06
ESR, median ± IQR	10 (7.0–17.5)	11 (4.3–20.8)	0.69
CRP, median ± IQR	2 (1–5)	3 (1–3)	0.58
DAS28, median ± IQR	2.3 (1.6–3.3)	2.2 (1.2–3.2)	0.78
ACPA+	16 (64%)	20 (83%)	0.20
RF+	20 (80%)	19 (79%)	> 0.99
DMARDs	18 (72%)	22 (92%)	0.14
Methotrexate	15 (60%)	21 (88%)	0.0507
Methotrexate (dose), median + IQR	15 (12.5–20)	20 (15–25)	0.10
Biologics	12 (48%)	9 (38%)	0.57
TNF inhibitors	6 (24%)	8 (33%)	0.54
Rituximab	3 (12%)	1 (4%)	0.61
NSAIDs	17 (68%)	13 (54%)	0.39
Corticosteroid use	3 (12%)	1 (4%)	0.61
Cardiovascular diseases	5 (20%)	5 (21%)	> 0.99
Previous malignancy	4 (16%)	8 (33%)	0.20
Lung disease	1 (4%)	1 (4%)	> 0.99
Never-smokers	9 (36%)	10 (42%)	0.77
Smokers	2 (8%)	1 (4%)	> 0.99
Ex-smokers	14 (56%)	13 (54%)	> 0.99

Table I show values at baseline. Statistical analyses were performed using the Fisher’s exact test or the Mann-Whitney U-test

### Randomization

We used stratified randomization with sex as a stratifying variable. A person not involved in intervention or examination conducted the randomization using sealed opaque envelopes and a computer generated sequence of allocation into one of two groups, exercise or control [15].

### Intervention

The exercise intervention group (*n* = 24) followed an exercise protocol of gym-based resistance and aerobic exercise performed at moderate- to high intensity (defined as 70–89% of maximum heart rate and 70–80% of 1 repetition maximum) 3 times a week, with person-centred guidance from physiotherapist. Both the intervention group and the active control group (*n* = 25) were encouraged to perform low-intensity daily physical activity plus home-based exercises for mobility, strength and balance without any weights or equipment 2 times a week. The exercise protocols and the person-centred approach are described in detail in Lange et al. 2019 [15].

### Maximal oxygen consumption measurement

Maximal oxygen consumption - VO_2_ max was measured by *cardiopulmonary exercise testing (CPET). Oxygen content of expired gas was measured during a bicycle ergometer test, using a metabolic chamber* (OxyCon, Jaeger, Sollentuna, Sweden) and the maximal oxygen consumption per minute and kg body weight (relative VO_2_ max) was calculated (mlO_2_/min/kg).

### Clinical assessment and disease activity

All patients were examined by a physician and a physiotherapist at baseline and after 20 weeks. The examiners were blinded to group allocation. Height, weight and blood pressure were measured. Disease activity was determined by the composite scores disease activity score 28 (DAS28) and clinical disease activity index (CDAI). Disability was determined by the Health Assessment Questionnaire - Disability Index (HAQ-DI). Dynamic leg muscle strength was assessed using the *Sit To Stand* (STS) test [15]. At the end of the study, patients rated their change of health since the study start on the Patient Global impression of Change (PGIC) scale ranging from 1 (very much improved) to 7 (very much worse). The clinical variables are previously reported in [15].

### Collection of blood samples

At baseline and 20 weeks, peripheral blood was collected from the participants between 8 and 12 am. The 20-week sampling was collected at least 24 h after the last exercise session. C reactive protein (CRP) was analysed by the routine Clinical Chemistry lab, Sahlgrenska University Hospital, Gothenburg, Sweden.

### Isolation of peripheral blood mononuclear cells (PBMCs) and plasma

The blood cells were counted on a KX-21 N™ Automated Hematology Analyzer (Sysmex) and centrifuged at 800 x g for 10 min. Plasma was transferred to cryotubes and stored at − 80 degrees until analysis. PBMC were isolated using SepMate tubes (STEMCELL Technologies) and stored at − 150 degrees in FCS with 10% DMSO.

### Phenotypic characterization using flow cytometry

Thawed PBMC were washed in FACS buffer (PBS, 10% heat inactivated FBS, 1 mM EDTA) and blocked in FACS buffer with 0.5% mouse serum. Antibodies used in this study are listed in (Additional file 1). Intracellular staining was performed using the Foxp3 Transcription Factor Staining Buffer Set (eBioscience). Cell samples from one individual pre and post exercise were thawed and stained at the same time and acquired using the same flow cytometric settings on a BD FACSVerse™. Gating strategies are shown for Tregs in (Fig. 2a-c), Bregs and MDSCs in (Additional file 2). All phenotypic characterizations were performed using FlowJo version 9.9.4. (Tree Star, Ashland, Oregon). Identical gates were set for every patient sample pair (pre and post exercise). The absolute number of Tregs was calculated by multiplying the frequency of Tregs contained within the lymphocyte gate by the number of lymphocytes per ml blood, that was determined in whole blood before PBMC isolation using a a KX-21 N™ Automated Hematology Analyzer (Sysmex).

### Determination of myokines in plasma

Myokines, including IL-10, were measured using a ProcartaPlex Human Myokine Panel (lot: 117980001, 121,573,002, eBioscience). Limits of quantification were set by the manufacturer as follows: IL-10 (1.94 pg/ml), BDNF (1.35 pg/ml), LIF (5.74 pg/ml), TNFα (8.03 pg/ml), IL-8 (2.47 pg/ml), IL-1RA (33 pg/ml), IL-15 (2.31 pg/ml), IL-6 (9.99 pg/ml). The samples were read on a Bio-Plex® 200 array reader (Bio-rad). IGF-1 was measured using a human IGF-1 duoset ELISA with plasma samples diluted 1:30 (R&D systems, detection limit 31 pg/mL). Concentrations below the detection limit and to low to be extrapolated were assigned a value of 0 pg/ml.

### T cell proliferation assay

Thawed PBMC were washed in cell culture medium. CD4+ T cells were isolated using negative isolation with Antibody Mix Human CD4 and depletion MyOne beads from Dynabeads® Regulatory CD4 + CD25+ T Cell Kit (Dynabeads). Cells were stained using CellTrace violet cell proliferation kit (Invitrogen) at a working concentration of 1.25 μM and a cell concentration of 1 ×  10^6^ cells /ml. CD4+ T cells were seeded at 1 × 10^5^ cells/well in a 96-well round bottom plate pre-coated with 1,5 μg/ml mouse anti-human CD3e (clone: OKT3, Invitrogen) in Complete medium (RPMI 1640 GlutaMAX, with 10% FCS, Non-Essential Amino Acids, 1 mM Sodium pyruvate, 50 μM β-mercaptoethanol, 100 U/ml Penicillin-Streptomycin (all from GIBCO)), with 1 μg/ml of mouse anti-human CD28 (clone: CD28.2, BD Pharmingen). After 4 days, supernatants were harvested and put in − 80 °C. Cells were stained with antibodies against cell surface antigens CD4, CD25 (Additional file 1) and 0.125 μg of the viability dye 7-AAD (BD Pharmingen) and aquired on a BD FACSLyric™. Analyses were performed using the proliferation tool in FlowJo version 9.9.4. (Tree Star, Ashland, Oregon). A LEGENDplex bead-based immunoassay was used to analyse the cytokines IL-2, IL-10, IFNγ and IL-17A in the supernatants. The assay was performed according to the manufacturer’s instructions, acquired on a BD FACSLyric™ and analysed using the Legendplex v7.1 software (BioLegend). Limits of quantification were set by the manufacturer as follows: IL-2 (1.4 pg/ml), IL-10 (0.9 pg/ml), IFNγ (1.3 pg/ml), IL-17A (2 pg/ml).

### Statistics

Statistical analyses were performed using GraphPad Prism (La Jolla, CA, USA). Wilcoxon signed-rank test was used to calculate the difference between continuous data obtained at baseline (pre) and after 20 weeks (post). The *Mann*-*Whitney U* test was used for comparisons of continuous variables, and the fisher’s exact test was used for comparisons of nominal variables, between the two groups. A ratio paired t-test was used to compare data from the T cell proliferation assay between samples collected at baseline (pre) and after 20 weeks (post). A spearman correlation test was used to analyse associations between the frequency of immune cell populations and clinical variables. *P*-values < 0.05 was considered significant.

## Results

### General physical effects of moderate- to high intensity exercise on health in older adults with RA

**Fig. 1:**
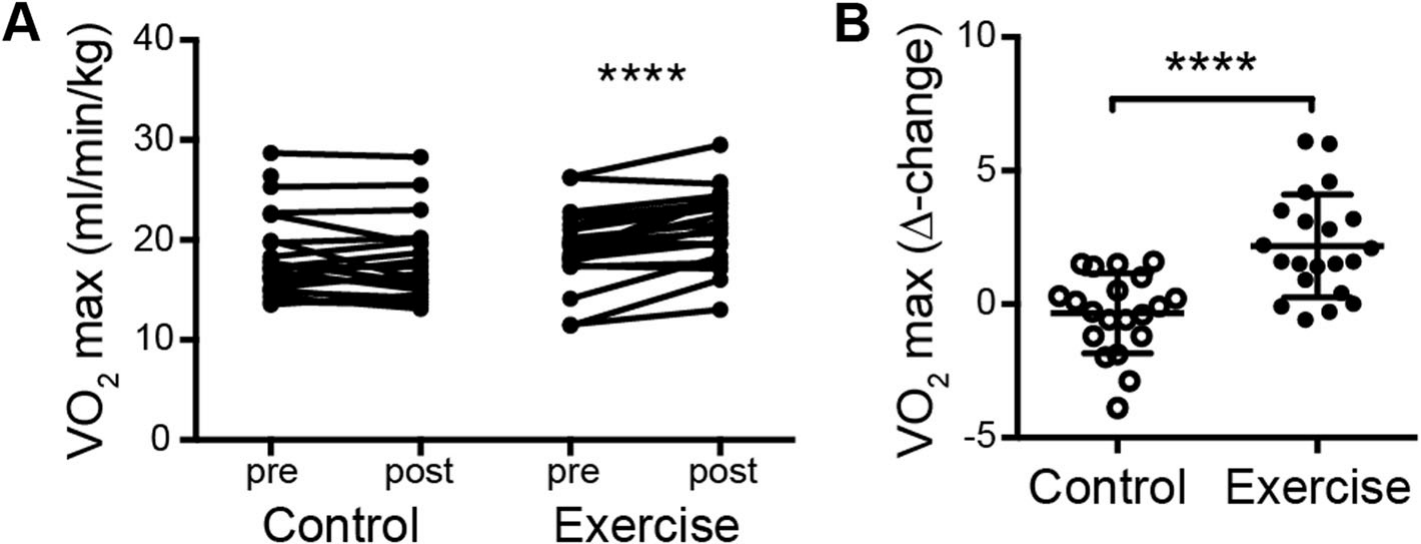
Changes in respiratory fitness in response to 20 weeks of exercise intervention in older adults with RA. Graphs showing **a**.the paired measurements (pre and post 20 weeks) of VO2 max (mlO_2_/min/kg) and **b**. the Δ-change value (between pre and post) in the control and exercise intervention group . *P* values were determined using Wilcoxon signed-rank test comparing data obtained at baseline (pre) and after 20 weeks (post). The *Mann*-*Whitney U* test was *used for comparison* between the two groups. *p ≤ 0.05 ** *p* ≤ 0.01 *** *p* ≤ 0.001 **** *p* ≤ 0.0001

Older adults (≥65 years) with RA were randomized to either 20-weeks of exercise intervention (*n* = 24) or control intervention (*n* = 25). There were no significant differences between the groups at baseline. Aerobic capacity, measured as the relative VO_2_ max, was assessed after 20 weeks of exercise or control intervention to validate the effect of the exercise program. As previously reported for the entire cohort [15], the relative VO_2_ max (Fig. 1a-b) and leg muscle strength, evaluated as sit-to-stand ability (Table 2), was improved within the intervention group, but not in the control group, in the Gothenburg-cohort. There was also a significant difference in Δ-change between groups (Table 2). The intervention group in the Gothenburg-cohort also improved their systolic blood pressure and BMI within the group, whereas there were no significant changes in CRP, N/L-ratio, DAS28, CDAI or the HAQ-DI within or between groups (Table 2 and [15]). Still, a significantly higher proportion of the intervention group rated PGIC as improved compared to the control group both in this subanalysis (Table 2) and in the entire cohort [15].

**Table 2:** Variables pre and post intervention

	Control		Pre-post p-value	Exercise		Pre-post p-value	Δ-change p-value
	Pre (0 weeks)	Post (20 weeks)		Pre (0 weeks)	Post (20 weeks)		
	*n* = 25	*n* = 24		*n* = 24	*n* = 24		
VO2 max (mlO_2_/min/kg)	17.2 (15.2–19.9)	16 (14.8–19-7)	0.45	19.3 (17.6–21.3)	21.5 (19.2–24.2)	< 0.0001	< 0.0001
STS (number)	21 (19–26)	24 (20–28)	0.02	23 (21–25)	27 (25–28)	< 0.0001	0.02
HAQ-DI	0.50 (0.25–0.94)	0.50 (0.25–0.97)	0.69	0.19 (0.0–0.84)	0.13 (0.0–0.72)	0.61	0.81
BMI	27.5 (24.8–30.4)	27.6 (24.6–29.8)	0.63	25.5 (21.4–28.0)	24.9 (21.3–27.9)	0.005	0.053
Systolic BP (mm Hg)	130 (123–145)	128 (120–134)	0.24	133 (130–140)	130 (120–134)	0.004	0.43
Diastolic BP (mm Hg)	80 (75–80)	80 (75–80)	0.92	80 (76–80)	80 (70–84)	0.51	0.77
CRP	2 (1–4)	2 (1–4)	0.65	2.5 (1–5.75)	2 (1–5.75)	0.36	0.73
N/L ratio	1.79 (1.31–2.76)	2.09 (1.29–2.78)	0.88	2.11 (1.53–2.96)	2.03 (1.38–2.75)	0.80	0.56
DAS28	2.27 (1.56–3.26)	2.12 (1.62–2.73)	0.63	2.23 (1.24–3.22)	2.04 (1.50–2.79)	0.47	0.88
CDAI	3.9 (2.6–6.6))	3.8 (2.0–6.9)	0.30	3.2 (2.0–7.1)	2.2 (0.7–8.0)	0.09	0.31
PGIC	–	3 (2.3–3.8)		–	2 (2.0–3.0)		0.02

*Abbrevations*: *VO*_*2*_*max* maximal oxygen consumption, *STS* Sit To Stand test, *HAQ-DI* Health Assessment Questionnaire Disability Index, *BMI* Body Mass Index, *BP* blood pressure, *CRP* C-reactive protein, *N/L ratio* Neutrophil/lymphocyte ratio, *DAS28* Disease Activity Score in 28 joints *CDAI* Clinical Disease Activity Index, *PGIC* Patient Global Impression of Change. Values are shown as median ± IQR. P-values are calculated using the Wilcoxon signed rank test for matched pre- and post samples or the Mann-Whitney test to compare the Δ-change between groups

### Moderate- to high intensity exercise reduces the frequency of regulatory T cells in older adults with RA

**Fig. 2:**
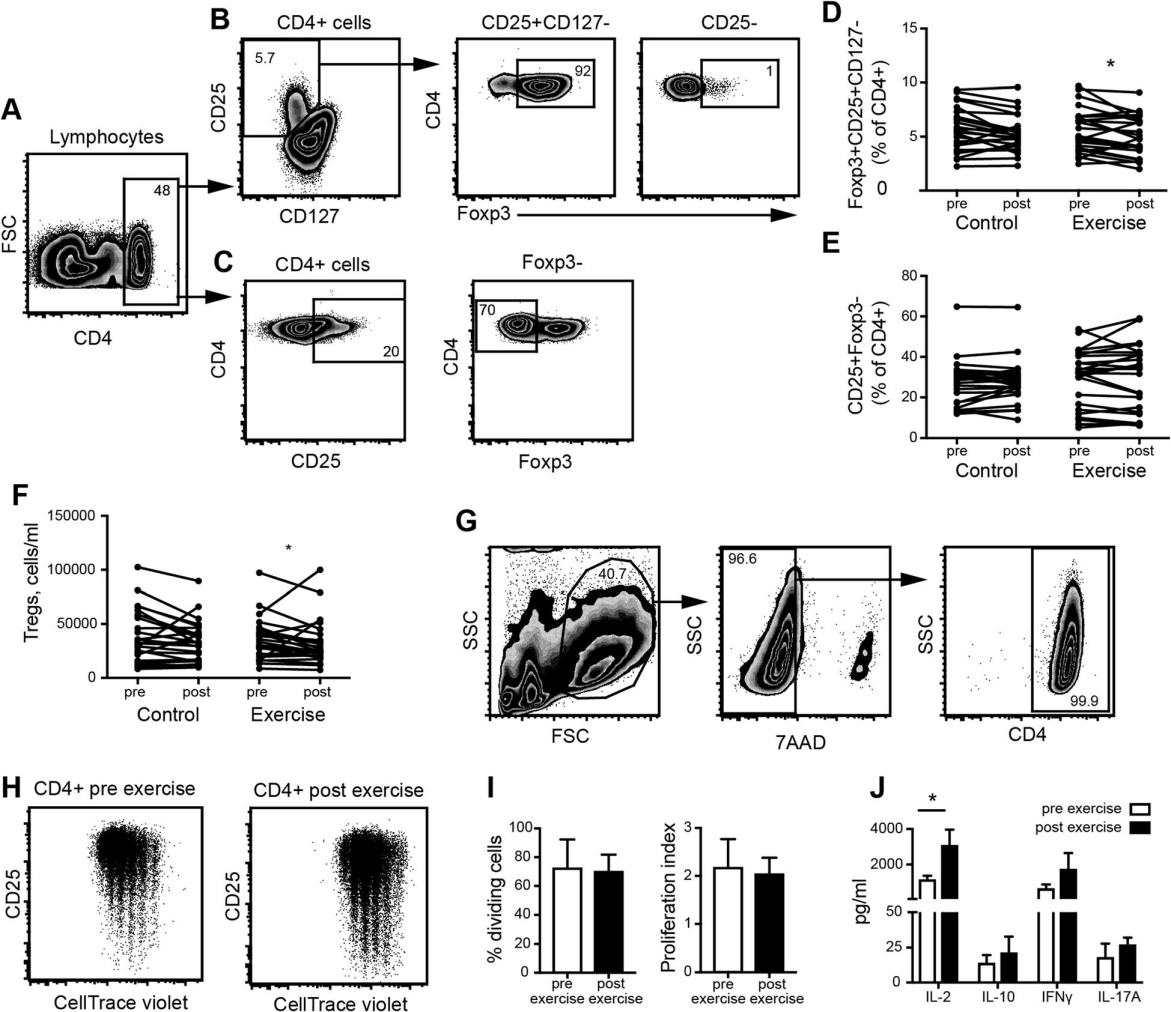
Changes in peripheral blood regulatory T-cells (Treg) frequencies in response to 20 weeks of exercise intervention in older adults with RA. **a.** CD4+ cells were gated from the total number of lymphocytes and then **b.** further gated into CD127-CD25+. The Foxp3+ Tregs were identified within the CD127-CD25+ and the gate was set based on lack of expression in the CD4 + CD25- cells. **c.** effector T cells were identified by a CD25+ gate, then a Foxp3- gate set adjacent to the Foxp3+ gate. Paired measurements of **d.** CD127-CD25 + Foxp3+ Tregs, **e.** CD25 + Foxp3- effector T cells frequencies in blood and **f.** absolute number of Tregs/ml, pre and post 20 weeks of control (*n* = 24) or exercise (*n* = 24) intervention. Frequencies are related to the CD4+ gate. **g**. Flow cytometry gating of proliferating CD4+ T cells after 4 days of stimulation with anti-CD3/anti-CD28 and **h**. CellTrace violet staining of CD4+ T cells from matched samples collected pre (*n* = 4) and post exercise (*n* = 4) with **i.** graphs showing % dividing cells and proliferation index analysed by the FlowJo proliferation analysis tool and **j.** cytokines in cell culture supernatants. *P* values were determined using Wilcoxon signed-rank test comparing data from samples collected at baseline (pre) and after 20 weeks (post). The *Mann*-*Whitney U* test was *used for comparison* between the two groups. A ratio paired t-test was used to compare data from the T cell proliferation assay between samples collected at baseline (pre) and after 20 weeks (post). *p ≤ 0.05 ** *p* ≤ 0.01 *** *p* ≤ 0.001 **** *p* ≤ 0.0001

To study the effect of moderate- to high intensity exercise on anti-inflammatory immune cell populations in older adults with RA, we first assessed the Treg subset in peripheral blood collected before and after 20 weeks of exercise. The frequency of circulatory CD4 + Foxp3 + CD25 + CD127- Tregs was decreased in the intervention group after 20 weeks of exercise (Fig. 2d). Also, the absolute number of Tregs was reduced in the intervention group but not in the control group after 20 weeks of exercise (Fig. 2f). We further analysed whether a reduction in Tregs would influence the proportion of CD25 + Foxp3- effector T cells, but no differences were found within or between the groups (Fig. 2e). The decrease in Tregs did not lead to increased proliferation of total CD4+ T cells (Fig. 2 g-i). There was a significant increase in IL-2 (Fig. 2j) in the supernatants after stimulation with anti-CD3/CD28 for 4 days, when comparing paired patient samples from the intervention group collected before (*n* = 4) and after exercise (*n* = 4) and that had Δchange in Tregs < − 1. The same tendency was seen with respect to production in IFNγ, IL17A or IL-10, however without statistical significance. There were no changes in the cell counts of total white blood cells, neutrophils, lymphocytes or the proportions of CD3+ T cells, CD4+ T cells and CD8+ T cells (Table 3). No correlation was found between Δ-change in Tregs and the Δ-change in DAS28, CRP or BMI.

**Table 3:** Number and frequency of peripheral blood cells pre and post intervention

	Control			Exercise		
	Pre (0 weeks)	Post (20 weeks)	Pre-post *p*-value	Pre (0 weeks)	Post (20 weeks)	Pre-post *p*-value
WBC (× 10^6^/ml) (mlO_2_/min/kg)	5.3 (4.2–6.5)	5.0 (4.2–5.7)	0.33	5.1 (4.3–6.1)	4.7 (4.1–5.9)	0.41
Neutrophils (× 10^6^/ml)	2.9 (2.3–4.0)	2.9 (2.4–3.6)	0.58	3.0 (2.6–3.7)	2.9 (2.3–3.6)	0.69
Lymphocytes (× 10^6^/ml)	1.6 (1.2–2.0)	1.6 (1.1–1.9)	0.31	1.7 (1.1–2.1)	1.4 (1.1–1.9)	0.19
% CD3 PBMC	44 (37–53)	45 (40–55)	0.44	50 (40–63)	47 (40–57)	0.24
% CD4+ of CD3+ PBMC	67 (51–78)	63 (49–76)	0.37	63 (53–77)	63 (56–77)	0.36
% CD8+ of CD3+ PBMC	29 (16–39)	32 (19–44)	0.27	27 (17–41)	29 (19–37)	0.43
% CD19+ lymphocytes	9.6 (7.5–16)	11 (8.4–14)	0.58	10 (6.1–13)	9.6 (5.8–14)	0.57
% MDSCs of PBMC	1.7 (1.1–2.2)	1.6 (1.2–2.7)	0.38	2.2 (1.2–2.9)	1.9 (1.1–3.3)	0.88

Values are shown as median ± IQR. P-values are calculated using the Wilcoxon signed rank test

### Moderate- to high intensity exercise reduces the frequency of regulatory B cells and serum levels of IL-10 in older adults with RA

**Fig. 3:**
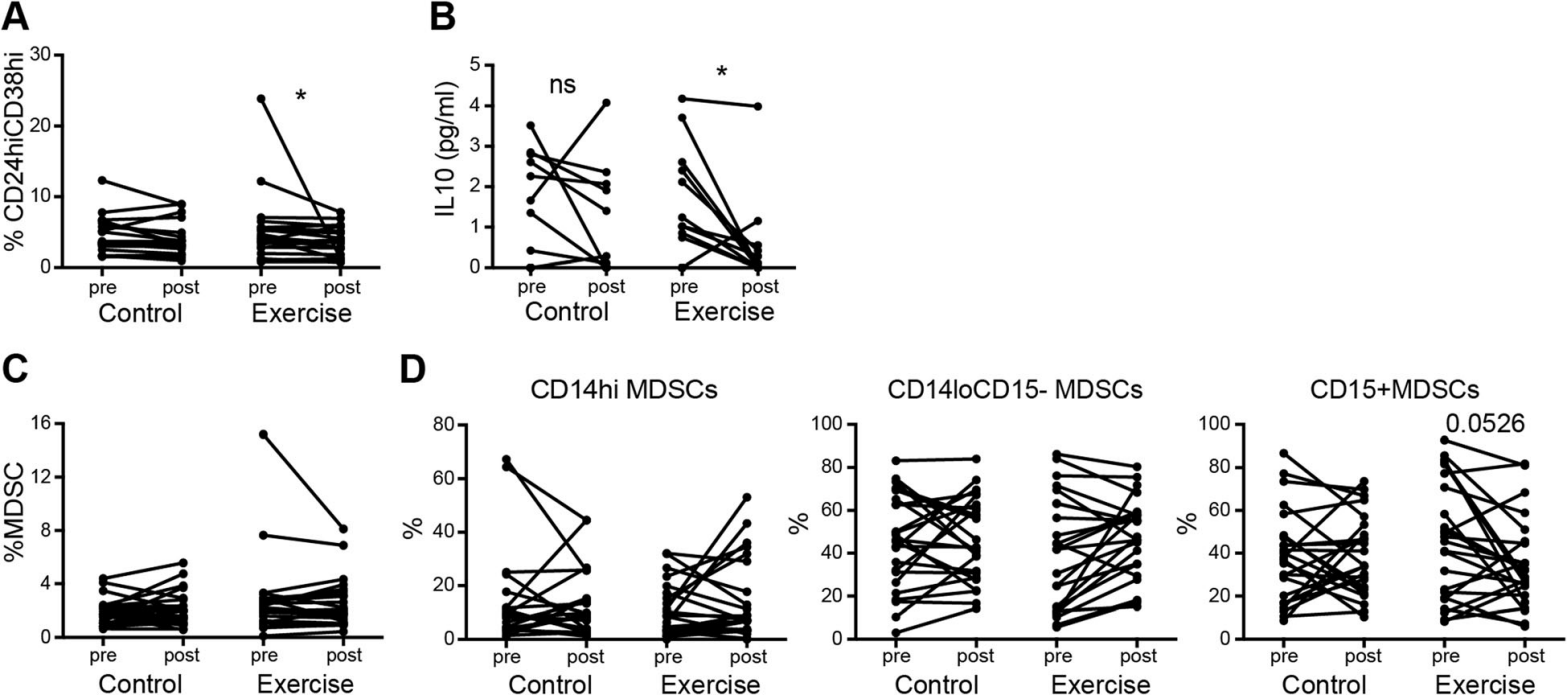
Changes in CD24hiCD38hi B cells (Bregs), IL-10 and MDSCs in peripheral blood in response to 20 weeks of exercise intervention in older adults with RA. **a.** Paired measurements of the CD24hiCD38hi B cell frequencies (related to the CD19+ gate) in blood pre and post 20 weeks of control (*n* = 20) or exercise (*n* = 22) intervention and **b.** IL-10 (pg/ml) in plasma, pre and post 20 weeks of control (*n* = 24) or exercise (*n* = 24) intervention. **c.** Paired measurements of the frequencies of CD33 + CD11b + MDSCs and **d.** MDSC subsets: CD14hi, CD14loCD15- and CD15+ in blood, pre and post 20 weeks of control (*n* = 24) or exercise (*n* = 24) intervention. *P* values were determined using Wilcoxon signed-rank test comparing data obtained at baseline (pre) and after 20 weeks (post). The *Mann*-*Whitney U* test was *used for comparison* between the two groups. *p ≤ 0.05 ** p ≤ 0.01 *** p ≤ 0.001 **** p ≤ 0.0001

We further assessed the effects of moderate- to high intensity exercise on the regulatory CD24hiCD38hi B cell population (Bregs). We observed a reduced frequency of Bregs after 20 weeks in the exercise intervention group but not in the control group (Fig. 3a). There was no change in the percentage of the total CD19+ population (Table 3). The decrease in regulatory blood lymphocyte populations after 20 weeks of exercise intervention was also accompanied by lower levels of IL-10 in plasma in the intervention group but not in the control group (Fig. 3b). There were no changes in the levels of other measured myokines after exercise intervention (Additional file 3). No correlation was found between Δ-change in Bregs to the Δ-change in DAS28, CRP or BMI. A weak positive correlation was found between Δ-change in IL-10 and Δ-change CRP (r = 0.365, *p* = 0.0116), but no correlation was found between the Δ-change in IL-10 and Δ-change in DAS28 or BMI.

### Moderate- to high intensity exercise does not affect the frequency of myeloid derived suppressor cells in older adults with RA

There was no difference within groups or between groups after the intervention period with respect to frequency of the innate anti-inflammatory MDSCs within the mononuclear cell population (Fig. 3c). We observed the presence of three different cell populations within this gate that could be further distinguished by the surface expression of CD14 and CD15. The proportion of CD15+ MDSCs showed a slight tendency to decrease, although not significant (Fig. 3d).

### The effect of moderate- to high intensity exercise on the Treg population differs in women and men

**Fig. 4:**
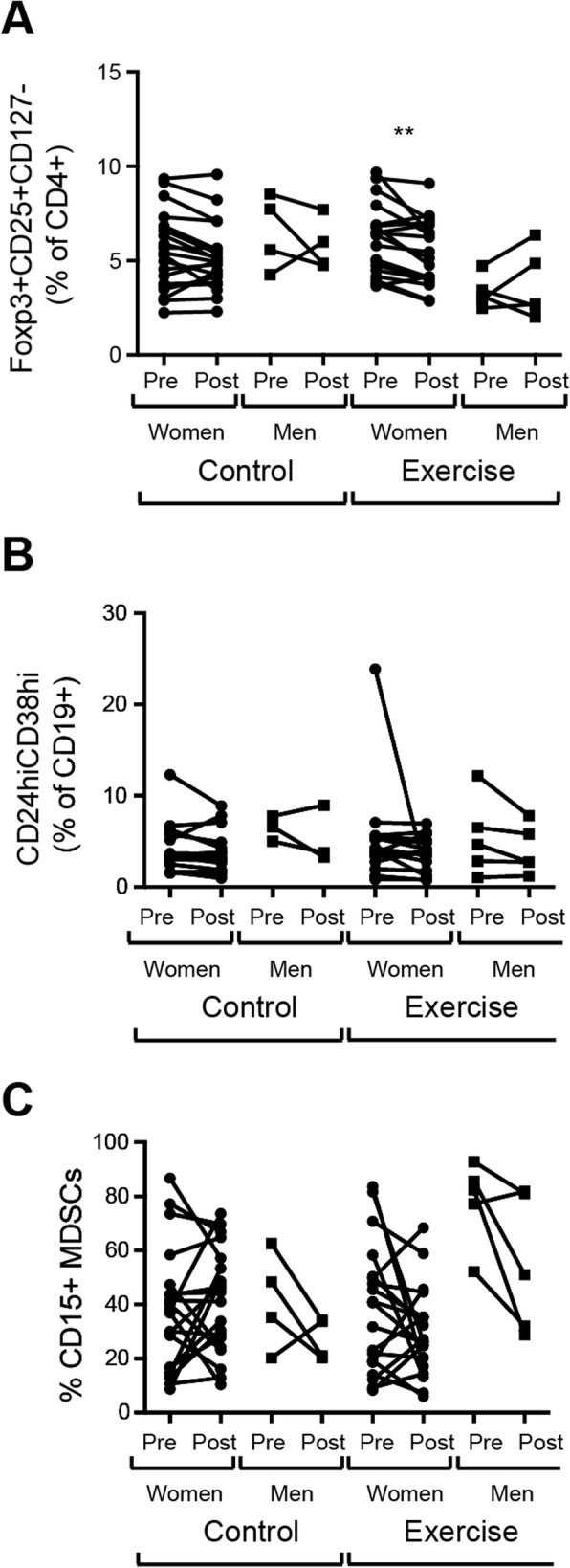
Changes in regulatory immune cell population frequencies in women and men in response to exercise intervention in older adults with RA. **a.** Paired measurements of CD127-CD25 + Foxp3+ Treg frequencies **b.** CD24hiCD38hi B cell frequencies and **c.** CD15+ MDSC frequencies in blood from women and men (pre and post 20 weeks) in the control (*n* = 24) and exercise (*n* = 24) intervention group respectively. *P* values were determined using Wilcoxon signed-rank test comparing data obtained at baseline (pre) and after 20 weeks (post). *p ≤ 0.05 ** *p* ≤ 0.01 *** *p* ≤ 0.001 **** *p* ≤ 0.0001

By dividing the participants by sex, we found that females in the intervention group responded to the moderate- to high intensity exercise by a reduced frequency of Tregs whereas males did not. There was no change in Tregs in the control group neither in females nor males (Fig. 4a). There was no sex difference in the response to moderate- to high intensity exercise with respect to Bregs (Fig. 4b) or CD15+ MDSCs (Fig. 4c).

## Discussion

In this report, we show that moderate- to high intensity exercise decreases Tregs and Bregs, as well as the production of IL-10 in older adults with RA, and that the effect on Tregs was only seen in females. To our knowledge this is the first study to demonstrate an effect of moderate- to high intensity exercise intervention on regulatory cell populations, i.e. peripheral Tregs and Bregs in older adults with RA.

As previously reported, a 20-week moderate- to high intensity exercise intervention clearly improved aerobic capacity as well as leg muscle strength in older adults with RA [15]. In healthy individuals the effects of exercise on the frequencies of Treg in peripheral blood differ between studies, possibly due to factors like age, time of sampling with respect to the time of exercise and the intensity [17–20]. In young elite athletes, a higher VO_2_ max was associated with an increase in both frequency and number of Tregs [17], while in marathon runners with a mean age of 38 years, there was a sustained post-race decrease in Tregs [18]. The time window between performed exercise and blood sampling needs to be taken into consideration as there are well documented transient responses to acute exercise [18, 19, 21]. However, we believe that more than 24 h between exercise and blood sampling is enough to reduce the most acute effects [19]. Possible mechanisms for the reduced Treg frequencies following exercise intervention are redistribution from the blood to other tissue [22], reduced peripheral differentiation of Tregs and increased apoptosis [6, 23]. We did not sample synovial fluid or tissue in this study, which is a limitation as these compartments often contain raised proportion of Tregs [7]. It would have been interesting to know if moderate- to high intensity exercise also reduced Tregs in the joint or if we would have seen a redistribution of Tregs to the joints. In this study, only the female participants in the intervention group responded with decreased Treg frequencies, which could be due to a hormonal influence [17] however there are few males, which is limitation and might influence the results.

Meta-analysis have shown that in RA-patients, the proportion of CD25 + Foxp3 positive Tregs in circulation are lower than in healthy and that lower frequencies are associated with active RA [7]. However, in our study we could not find any signs of increased inflammation or augmented disease after exercise despite lower Treg proportions. The reduction of Tregs after exercise did not seem to affect the proliferative response of T cells, nor production of IL-10, IFNγ and IL17A, despite lower IL-2 consumption. However, there are clear limitations of the anti-CD3/anti-CD28 assay, as it can only test bystander suppression by Tregs.

In the intervention group, we found a significant decrease in Bregs and IL-10. In healthy elderly, there is both a numerical and functional deficit of Bregs, associated with increased levels of autoantibodies including rheumatoid factor [9] and in patients with RA the immunosuppressive capacity [8, 24] of these cells appear reduced. Previous investigators found that Breg frequencies inversely correlated with inflammation in RA [24, 25]. Our results show that, despite the reduced Breg frequency after moderate- to high intensity exercise this was not associated with increased systemic inflammation as evidenced by stable CRP levels. A limitation is that we did not analyse the CD19 + IL-10+ B cells and therefore subsets of B cells may be missed. Also, the plasma levels of cytokines, including IL-10, were very low to undetectable in some of the samples, which gives less statistical power.

MDSCs, and in particular CD15+ MDSCs accumulate in peripheral blood with age [10]. This subset is almost undetectable in young healthy donors and accumulation of MDSCs may increase the susceptibility to infections, cancer and reduce response to vaccines [11]. However, moderate- to high intensity exercise did not significantly reduce MDSC numbers or frequency, though there was a trend towards an effect.

The lack of effects of exercise on disease activity in the intervention group is unexpected, as several studies have reported benefits of increased physical activity for DAS scores in patients with RA [13, 26]. This may be explained by the nature of our study cohort. A majority of the participants in this study were in remission or had low disease activity. We speculate that the exercise has a rejuvenating effect, in this case curbing the regulatory arm in order to improve healthy immune function. Initiation of regular exercise can indeed improve innate bactericidal function in older adults with RA [26]. This is in line with the fact that significantly more patients in the intervention group ranked their global impression of health (PGIC) higher at both 20 and 52 weeks compared to the control group, at the same time as there were no differences between the groups with respect to disease activity [15]. These findings suggested the moderate to high intensity exercise is beneficial in older patients with RA, and the reduction of regulatory immune cells in circulation does not influence the long-term outcome of disease.

## Conclusion

The results from this study show that moderate- to high intensity exercise in older adults with RA reduced the serum levels of IL-10 as well as the frequency of Tregs and Bregs, i.e. regulatory adaptive immune cell populations in the circulation. The effects on Tregs were more pronounced in the female participants. This reduction was not accompanied by a change in disease activity score but with an improvement in the patient’s own perceived health.

## Supplementary information

**Additional file 1 Supplementary Table I**. Flow cytometry antibody panels

**Additional file 2 Supplementary Fig. 2. A**. Gating of lymphocytes from PBMC singlets was done in a SSC versus FSC plot. B cells were identified by their expression of CD19+. Regulatory B cells were identified by their expression of CD24 and CD38 and defined as CD24hiCD38hi. Patients that was treated with rituximab < 1 year before inclusion or during the ongoing study, consequentially lacked B cells and were thus omitted from the flow cytometric **B** cell analysis. The total number of patients omitted from the B cell analysis was 6, 2 from the exercise group and 4 from the control group. B. Myeloid derived suppressor cells were gated from the mononuclear cells negative for HLA-DR and CD56. The MDSCs were then identified as being positive for CD33 and CD11b. The three distinct MDSC populations were separated by their expression of CD14 and CD15, namely the CD15+, CD14loCD15- and CD14hi. Identical gates were set for every patient sample pair (pre and post exercise). All phenotypic characterizations were performed using FlowJo version 9.9.4. (TreesStar, Ashland, Oregon).

**Additional file 3 Supplementary Fig. 3**. The concentrations of BDNF, LIF, TNFα, IL-8, IL1-RA, IL-15. IL-6 and IGF-1 in plasma samples, pre and post 20 weeks of control (*n* = 24) or exercise (*n* = 24) intervention. The lower level of quantification is marked with a dotted line. *P* values were determined using Wilcoxon signed-rank test comparing data obtained at baseline (pre) and after 20 weeks (post).

## Data Availability

The datasets used and analysed during the study are available from the corresponding author on reasonable request.

## Abbreviations

RA: Rheumatoid arthritis
Tregs: Regulatory T cells
Bregs: Regulatory B cells
MDSCs: Myeloid derived suppressor cells
DAS28: Disease Activity Score 28
CRP: C-reactive protein
PGIC: Patient’s global impression of change
VO_2_ max: Maximal oxygen consumption
CDAI: Clinical disease activity index
HAQ-DI: Health Assessment Questionnaire - Disability Index
PBMC: Peripheral blood mononuclear cells
IL-10: Interleukin 10
