# Supplementary material for: Moderate- to high intensity aerobic and resistance exercise reduces peripheral blood regulatory cell populations in older adults with rheumatoid arthritis
Source: Immun Ageing. 2020 May 16;17:12. doi: 10.1186/s12979-020-00184-y (PMC7229606; doi:10.1186/s12979-020-00184-y)
Supplement: Supplementary file 1 — Additional file 1 Supplementary Table I. Flow cytometry antibody panels [file 12979_2020_184_MOESM1_ESM.pdf]

Supplementary table I: Flow cytometry antibody panels

Panel 1

Host	Target	Clone	Conjugate	Manufacturer	Dilution
mouse	CD4	SK3	APC-H7	BD Biosciences	1:33
mouse	CD25	2A3	APC	BD Biosciences	1:50
mouse	CD127	HIL-7R-M21	FITC	BD Biosciences	1:16
rat	Foxp3	PCH101	PE	eBioscience	1:40

Panel 2

Host	Target	Clone	Conjugate	Manufacturer	Dilution
mouse	CD19	HIB19	V500	BD Biosciences	1:40
mouse	CD24	ML5	PECy7	BD Biosciences	1:40
mouse	CD38	HB7	APC-H7	BD Biosciences	1:40
mouse	CD27	L128	PE	BD Biosciences	1:40
mouse	IgD	IA6-2	FITC	BD Biosciences	1:50
mouse	CD11c	3.9	BV421	Biolegend	1:20
mouse	CD21	Bu32	PerCp-Cy5.5	Biolegend	1:50
mouse	CD10	HI10a	APC	BD Biosciences	1:50

Panel 3

Host	Target	Clone	Conjugate	Manufacturer	Dilution
mouse	CD33	WM-53	PE-Cy7	eBioscience	1:100
mouse	CD11b	ICRF44	PerCp-Cy5.5	Biolegend	1:50
mouse	HLA-DR	G46-6	APC	BD Biosciences	1:20
mouse	CD56	NCAM16.2	FITC	BD Biosciences	1:20
mouse	CD14	MφP9	APC-H7	BD Biosciences	1:33
mouse	CD15	W6D3	BV510	BD Biosciences	1:20
mouse	CD16	3G8	BV421	BD Biosciences	1:100
hamster	KLRG1	2F1	PE	eBioscience	1:20

Panel 4

Host	Target	Clone	Conjugate	Manufacturer	Dilution
mouse	CD3	UCHT1	BV421	BD Biosciences	1:50
mouse	CD4	SK3	APC-H7	BD Biosciences	1:33
mouse	CD8	SK1	V500	BD Biosciences	1:100
mouse	CD28	CD28.2	APC	BD Biosciences	1:100
mouse	CD45RA	L48	FITC	BD Biosciences	1:100
rat	CCR7	3D12	PECy7	BD Biosciences	1:40
hamster	KLRG1	2F1	PE	eBioscience	1:20
mouse	CD57	HNK-1	bitotin (streptavidin PerCp)	Biolegend	1:200
