# Supplementary material for: Moderate- to high intensity aerobic and resistance exercise reduces peripheral blood regulatory cell populations in older adults with rheumatoid arthritis
Source: Immun Ageing. 2020 May 16;17:12. doi: 10.1186/s12979-020-00184-y (PMC7229606; doi:10.1186/s12979-020-00184-y)
Supplement: Supplementary file 2 — Additional file 2 Supplementary Fig. 2. A. Gating of lymphocytes from PBMC singlets was done in a SSC versus FSC plot. B cells were identified by their expression of CD19+. Regulatory B cells were identified by their expression of CD24 and CD38 and defined as CD24hiCD38hi. Patients that was treated with rituximab < 1 year before inclusion or during the ongoing study, consequentially lacked B cells and were thus omitted from the flow cytometric B cell analysis. The total number of patients omitted from the B cell analysis was 6, 2 from the exercise group and 4 from the control group. B. Myeloid derived suppressor cells were gated from the mononuclear cells negative for HLA-DR and CD56. The MDSCs were then identified as being positive for CD33 and CD11b. The three distinct MDSC populations were separated by their expression of CD14 and CD15, namely the CD15+, CD14loCD15- and CD14hi. Identical gates were set for every patient sample pair (pre and post exercise). All phenotypic characterizations were performed using FlowJo version 9.9.4. (TreesStar, Ashland, Oregon). [file 12979_2020_184_MOESM2_ESM.pdf]

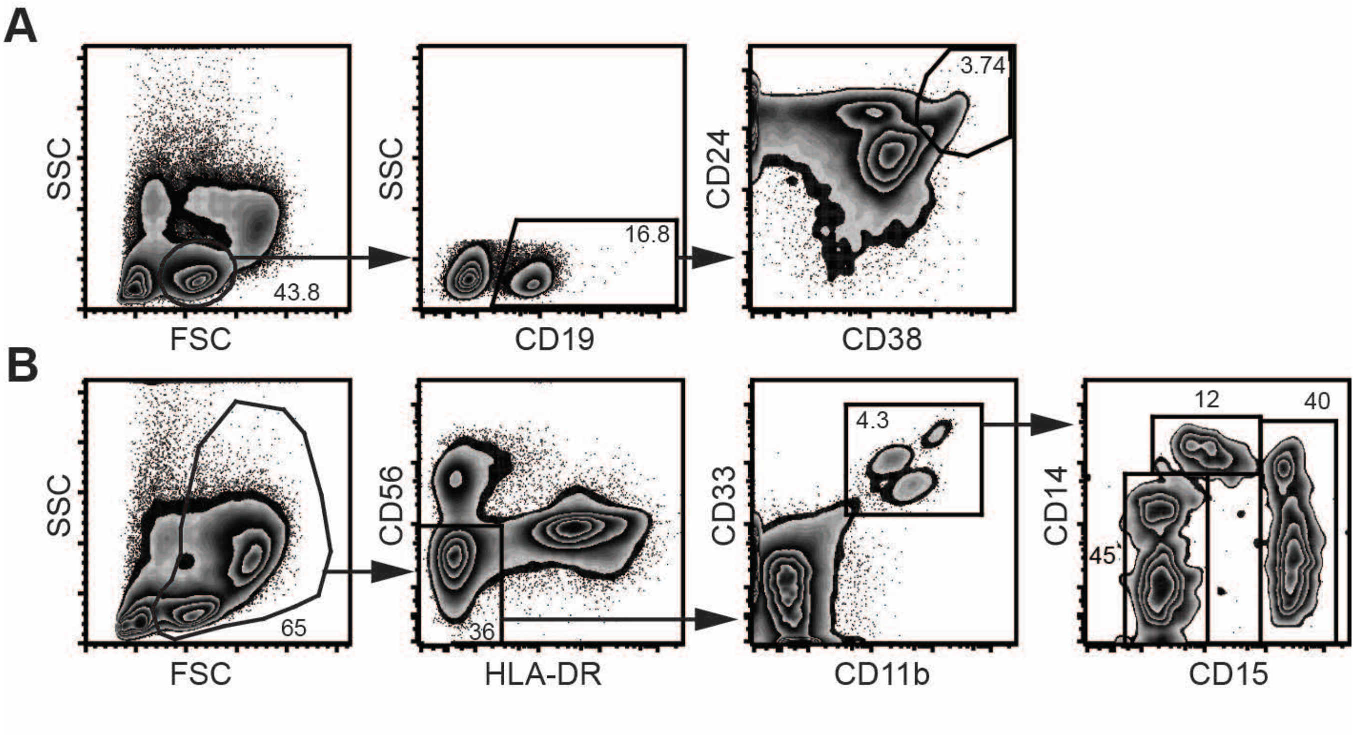

Supplementary figure 2

A. Gating of lymphocytes from PBMC singlets was done in a SSC versus FSC plot. B cells were identified by their expression of CD19+. Regulatory B cells were identified by their expression of CD24 and CD38 and defined as CD24hiCD38hi. Patients that was treated with rituximab <1 year before inclusion or during the ongoing study, consequentially lacked B cells and were thus omitted from the flow cytometric B cell analysis. The total number of patients omitted from the B cell analysis was 6, 2 from the exercise group and 4 from the control group. **B.** Myeloid derived suppressor cells were gated from the mononuclear cells negative for HLA-DR and CD56. The MDSCs were then identified as being positive for CD33 and CD11b. The three distinct MDSC populations were separated by their expression of CD14 and CD15, namely the CD15+, CD14loCD15- and CD14hi. Identical gates were set for every patient sample pair (pre and post exercise). All phenotypic characterizations were performed using FlowJo version 9.9.4. (TreesStar, Ashland, Oregon).
