# Supplementary material for: Moderate- to high intensity aerobic and resistance exercise reduces peripheral blood regulatory cell populations in older adults with rheumatoid arthritis
Source: Immun Ageing. 2020 May 16;17:12. doi: 10.1186/s12979-020-00184-y (PMC7229606; doi:10.1186/s12979-020-00184-y)
Supplement: Supplementary file 3 — Additional file 3 Supplementary Fig. 3. The concentrations of BDNF, LIF, TNFα, IL-8, IL1-RA, IL-15. IL-6 and IGF-1 in plasma samples, pre and post 20 weeks of control (n = 24) or exercise (n = 24) intervention. The lower level of quantification is marked with a dotted line. P values were determined using Wilcoxon signed-rank test comparing data obtained at baseline (pre) and after 20 weeks (post). [file 12979_2020_184_MOESM3_ESM.pdf]

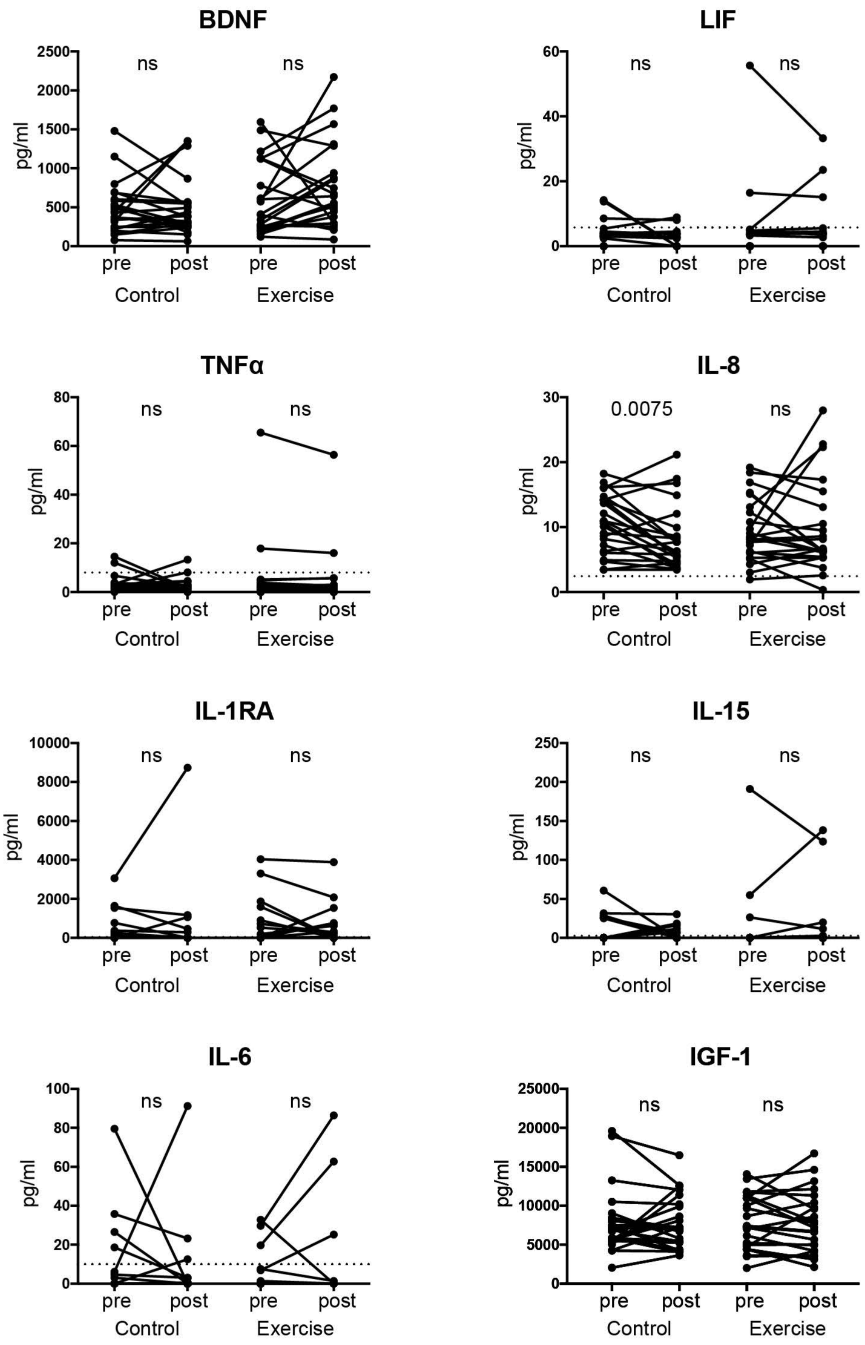

Supplementary figure 3

The concentrations of BDNF, LIF, TNFα, IL-8, IL1-RA, IL-15. IL-6 and IGF-1 in plasma samples, pre and post 20 weeks of control (n=24) or exercise (n=24) intervention. The lower level of quantification is marked with a dotted line. *P* values were determined using Wilcoxon signed-rank test comparing data obtained at baseline (pre) and after 20 weeks (post).
